# The Chinese Ebola Diagnostic and Treatment Center in Liberia as a model center

**DOI:** 10.1038/emi.2015.71

**Published:** 2015-11-25

**Authors:** Xiaowei Xu, Jianrong Huang, Haiwei Yang, Hai Xin, Yong Zhang, Qing Mao, Jianqi Lian

**Affiliations:** 1 Collaborative Innovation Center for Diagnosis and Treatment of Infectious Diseases; State Key Laboratory for Diagnosis and Treatment of Infectious Disease, First Affiliated Hospital, Zhejiang University School of Medicine, Hangzhou 310003, Zhejiang Province, China; 2 ChengDu Military General Hospital, Chengdu 610083, Sichuan Province, China; 3 Southwest Hospital, The First Affiliated Hospital, Third Military Medical University, Chongqing 400038, China; 4 Tangdu Hospital, The Second Affiliated Hospital, Forth Military Medical University, Shanxi 710038, Xi'an Province, China

## 

**Dear Editor,**

The Chinese Ebola treatment unit (China ETU) in Liberia is a field hospital installed by the Central Military Commission of China. Our medical team consisted of military troops and medical staff from local Chinese hospitals. The goal of our team was to fight Ebola hemorrhagic fever in Liberia while also preventing any of the medical team from getting infected. Safe and effective organization and management measures were essential for accomplishing these goals.

From November 2014 to March 2015, our medical team treated 125 outpatient and emergency cases, and only 77 of these patients were treated at the China ETU. During this time, we confirmed 10 cases of Ebola and cured six of these cases. The details of the Ebola epidemic in Liberia are listed in Figure 1.

Liberia is a country that lacks healthcare, education, and medical facilities. Moreover, the population suffers from a lack of awareness of disinfection and isolation procedures. We visited schools, factories, and Chinese enterprises and took firm, effective measures to disinfect and protect these facilities to reduce the risk of Ebola. For each facility, we repeated this action approximately 12 times. Furthermore, we aimed to increase public health awareness and improve disinfection and isolation procedures by hosting seven training courses on Ebola prevention and control, which trained 324 local clinicians and educators. We taught them proper hand-washing and disinfectant use to disinfect their houses and daily articles. We had close contact with 12 359 suspected or confirmed Ebola patients. We had contact with 409 blood samples, and contacted medical facilities 2556 times and individuals 9394 times. Despite the high level of contact, none of our medical team became infected with Ebola. During our service, we did not experience any nosocomial infections, accidents, or patient complaints, including medical malpractice. Our strategy for implementing a successful plan consisted of military management, a collaborative work environment, collective decision-making, informational management, and strong logistics.

## MILITARY MANAGEMENT

The medical team originated from 19 hospitals in China, with team members specialized in infection, respiratory diseases, intensive care, nursing, and maintenance (i.e., plumbers and chefs). All team members were placed on a rigid schedule and were required to obey set rules. Human and financial resources were controlled by a single management group. Despite their different backgrounds, all team members obeyed the assigned duties without complaint.

## COLLABORATIVE WORK ENVIRONMENT

To create structure, we placed our employees in the following different departments: Command, Outpatient Records, Observation, Treatment, Medical Technology Support, Health and Epidemic Prevention, and Logistics Support. Upon arrival in Liberia, we quickly realized the importance of an infection prevention and control team, and we consequently formed this department, which became responsible for a variety of tasks (e.g., personal prevention training and exercises and the supervision of medical staff). The department formulated a workflow and series of regulations, which were strictly implemented. Each employee took a seven-day personal prevention training course that included basic Ebola knowledge, the process of engaging Ebola patients, and the use of isolation methods (e.g., gowns and protective barriers).

The medical staff was required to use personal protective equipment prior to direct contact with patients: protective clothing, masks, hats, gloves, eye shields, face shields, and rubber boots (Supplementary Figure S1). Two employees worked together to take isolation gowns on and off and to aid and supervise each other. Furthermore, nurses in the control room viewed the entire process on a monitor and communicated warnings if necessary. The living quarters and work areas were staffed with gate keepers who were responsible for tracking incoming and outgoing people, taking temperatures, and disinfecting the soles of individuals' shoes. Anyone whose temperature exceeded 37.3°C was banned from the living quarters and work areas and was forced into quarantine. If the fever persisted and Ebola had not been ruled out, the individuals were quarantined in an appointed area within the China ETU, which can separate individuals suffering from fever from the patients and the medical staff until the temperature recovered or Ebola was ruled out.

## COLLECTIVE DECISION-MAKING

Because few infection specialist employees were available, we published a regulatory document on the treatment and care of Ebola patients. We required that each medical employee study and implement the methods in this document. In addition, our employees were educated on the symptoms, diagnoses, and treatments of other common infectious diseases in Africa. We listened to and collaborated with local experts with experience in preventing and treating Ebola and other infectious diseases in Africa. We worked with two types of specialists: those responsible for the diagnosis and treatment of Ebola and those specializing in Ebola prevention and control. When problems were encountered (i.e., how to identify treatment plans or how to create emergency plans), we held committee meetings that involved collective discussions and decisions.

## INFORMATIONAL MANAGEMENT

To reduce hospital infections and the risk of error, we created an informational management system. For example, we used a medical records system to manage medical histories, patient progress notes, and doctors' notes. We installed surveillance cameras and intercoms in the wards to monitor patients, employees, and facility operations. Continuous surveillance was used to reduce the risk of error and improve the quality of medical care. To promote communication among employees, we provided each employee with a mobile phone.

## STRONG LOGISTICS

Because we faced a shortage of supplies in Liberia, strong logistics were critical for completing tasks. We purchased our own generators, water purifiers, and routine items, such as tables and cabinets. The maintenance staff was responsible for multiple jobs and worked on call.

Although the space was small, we established a psychological counseling office and a reading room. We also organized company activities, including social events and sporting games. Furthermore, employees were encouraged to write their feelings and experiences to reduce psychological stress.

To work in Liberia, we were required to enforce strict Chinese military orders and show unquestioning obedience. These rules prevented our team from leaving the wards and living quarters in order to avoid theft and public security issues. Despite our input, the local military set the rules, which we obeyed for our own safety and success.

A proper knowledge of infection control and strict regulations were key strategies for achieving our ‘no infection' goal.^1^ China ETU was not an ordinary infectious disease laboratory. Instead, it was a laboratory with a mission to fight the Ebola epidemic. Protection measures were meticulous but required modifications that differed from standard US^2^ and Chinese guidelines.^3^ These modifications were based on both the African temperatures and formulated sterile conditions. Although we came into contact with suspected or confirmed Ebola patients more than 12 359 times, none of our medical staff was infected. We attributed our safety to the effectiveness of our modified protective measures.

Employee awareness needed to be increased via meetings, training, and other measures.^1,4^ To prevent infection, the group was required to collaborate.^5^ Supervision and strong communication were essential to create an environment that prevented or easily remedied negligence. The health and living conditions of the employees needed to be continuously controlled and evaluated. The reaction time to emergencies should be rapid, and emergencies should be met with a well-controlled strategy. Furthermore, a contingency plan should be implemented immediately in the case of unfortunate.^6^

## Figures and Tables

**Figure 1:**
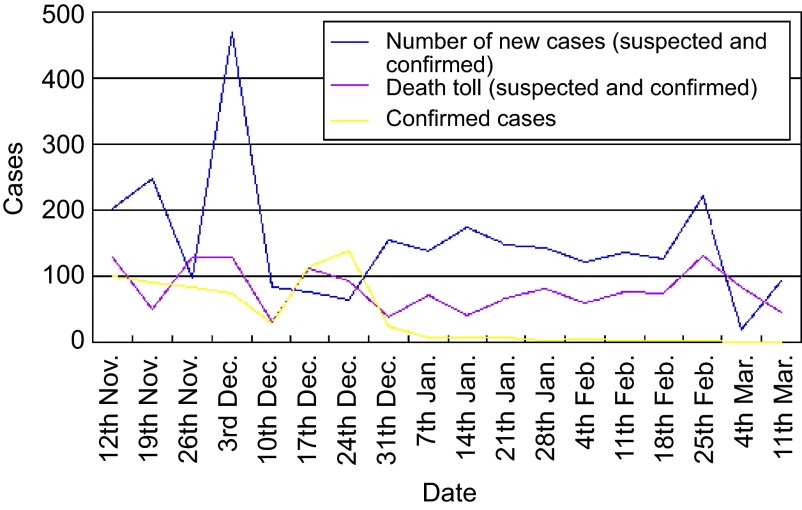
The epidemic situation of Ebola in Liberia (data from the World Health Organization).
