# Supplementary figures and images for: The Chinese Ebola Diagnostic and Treatment Center in Liberia as a model center
Source: Emerg Microbes Infect. 2015 Nov 25;4(11):e71–. doi: 10.1038/emi.2015.71 (PMC4661429; doi:10.1038/emi.2015.71)

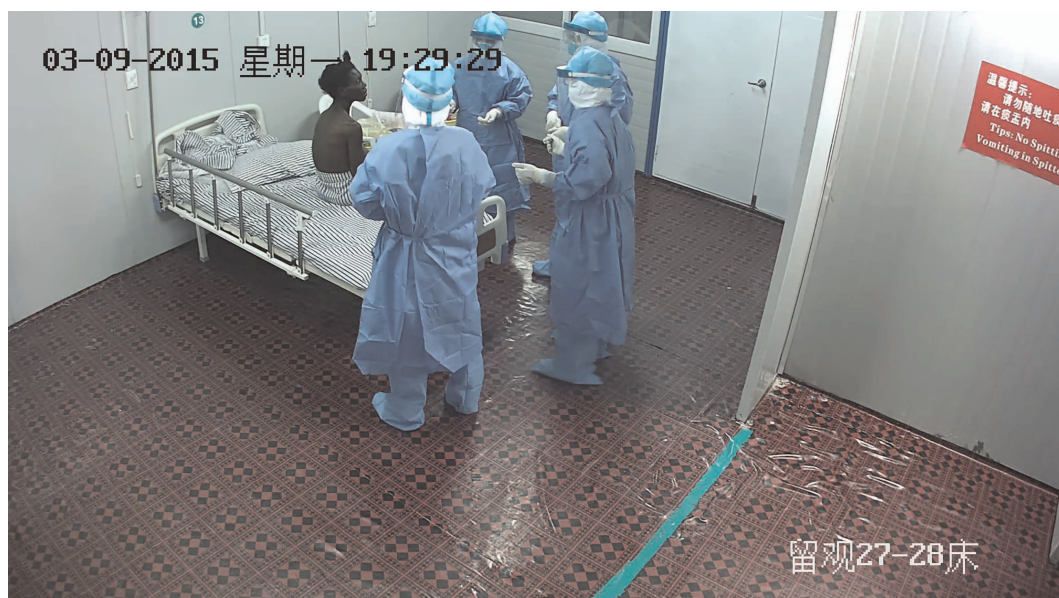

**Supplementary Figure S1** Proper protection and methods for treating Ebola patient.

Supplement: Supplementary Figure S1 [file emi201571x1.pdf]
